# Syphilitic Mediastinitis

**Published:** 1913-02

**Authors:** 


Syphilitic Mediastinitis.—Camille Lian and L. Baron of Paris, *Progres Medical,* November 16, 1912. The principal symptoms in a man of 30 were attacks of suffocation, spasms of the glottis, roaring in the ears, transient deafness, pulse sometimes 130, sometimes 40, at times arrhythmic; oedematous pharyngitis, enlarged cervical, axillary and inguinal nodes, liver two fingers' breadths below costal arch in mammillary line, left lobe filling epigrastric region to one finger's breadth above umbilicus, spleen perhaps slightly enlarged but not palpable, no ascites, inferior tegumentary epigastric vein of Braune, enlarged on each side so as to be plainly visible from inguinal regions to neck. The diagnosis was corroborated by an oblique radiographic examination, showing an irregular obscuration of the normally clear space between the cardio-aortic and vertebral shadows.

In a general discussion, various signs are mentioned: Broadbent's sign of pleuro-costal systolic retraction, in the precordium and below and outside of the scalpular angle; Kussmaul's paradoxic pulse; Wenckebach's sign, lower part of sternum not carried forward in inspiration as normally, on account of fibrous bands: Radoniicic's sign, that in the recumbent posture, the arterial pressure differs in the two sides, on account of pressure of the diaphragm, the difference disappearing when the patient sits up. On the whole, radiography is considered the most constant diagnostic method.

A female patient, aged 49, is mentioned as having also aneurysm of the aortic arch, with obliteration of the left brachiocephalic vein. Syphilitic tracheo-bronchitis and, especially in children, tuberculosis, with adenopathies, are mentioned as frequent complications.

